# Detection of diarrhoeagenic *Escherichia coli* in clinical and environmental water sources in South Africa using single-step 11-gene m-PCR

**DOI:** 10.1007/s11274-014-1690-4

**Published:** 2014-06-27

**Authors:** K. B. Omar, T. G. Barnard

**Affiliations:** Faculty of Health Sciences, Water and Health Research Centre, University of Johannesburg, Doornfontein, PO Box 17011, Johannesburg, 2028 South Africa

**Keywords:** Clinical/environmental samples, Diarrhoeagenic *E. coli*, Multiplex-PCR

## Abstract

*Escherichia coli* (*E. coli*) consists of commensal (ComEC) and diarrhoeagenic (DEC) groups. ComEC are detected using traditional culture methods. Conformational steps are performed after culturing if it is required to test for the presence of DEC, increasing cost and time in obtaining the results. The aim of this study was to develop a single-step multiplex polymerase chain reaction (m-PCR) that can simultaneously amplify genes associated with DEC and ComEC, with the inclusion of controls to monitor inhibition. A total of 701 samples, taken from clinical and environmental water sources in South Africa, were analysed with the optimised m-PCR which targeted the *eaeA*, *stx1*, *stx2*, *lt*, *st*, *ial*, *eagg*, *astA* and *bfp* virulence genes. The *mdh* and *gapdh* genes were included as an internal and external control, respectively. The presence of the external control *gapdh* gene in all samples excluded any possible PCR inhibition. The internal control *mdh* gene was detected in 100 % of the environmental and 85 % of the clinical isolates, confirming the classification of isolates as *E. coli* PCR positive samples. All DEC types were detected in varying degrees from the *mdh* positive environmental and clinical isolates. Important gene code combinations were detected for clinical isolates of 0.4 % *lt* and *eagg*. However, 2.3 % of *eaeA* and *ial*, and 8.7 % of *eaeA* and *eagg* were reported for environmental water samples. The *E. coli* *astA* toxin was detected as positive at 35 and 17 % in environmental isolates and clinical isolates, respectively. Interestingly, 25 % of the *E. coli* *astA* toxin detected in environmental isolates and 17 % in clinical isolates did not contain any of the other virulence genes tested. In conclusion, the optimised single-step 11-gene m-PCR reactions could be successfully used for the identification of pathogenic and non-pathogenic *E. coli* types. The m-PCR was also successful in showing monitoring for PCR inhibition to ensure correct reporting of the results.

## Introduction

*Escherichia coli* (*E. coli*) consists of both commensal (ComEC) and diarrhoeagenic (DEC) types. DEC not only indicate the presence of intestinal pathogens or parasites but also constitute a human health risk in themselves (Grabow et al. 2003; Kaper et al. 2004). At present, seven groups of pathogenic *E.* *coli* have been identified, of which five were selected for this study based on their importance for surface-water pathogenicity. The DEC types have been classified into the following: entero-pathogenic *E. coli* (EPEC), entero-toxigenic *E. coli* (ETEC), entero-haemorrhagic *E. coli* (EHEC), entero-aggregative *E. coli* (EAEC) and entero-invasive *E. coli* (EIEC) (Ashbolt 2004; Kaper et al. 2004). There are media available for the detection of specific EHEC 0157:H7 but traditional culture methods for *E. coli* were not designed for the detection of DEC (Iijima et al. 2007) but rather ComEC. Further conformational steps are thus required after culturing to distinguish the DEC from the ComEC which increases cost and time in producing the results. Diarrhoeagenic bacteria such as *Campylobacter jejuni*, *Salmonella enterica* serovar, *Shigella* spp., and *Vibrio* spp., can be readily isolated using selective plating media, with the exception of STEC 0157. Serotyping is the predominant means of differentiating pathogenic strains of *E. coli,* and phenotypic assays based on virulence characteristics can also identify DEC. Genotypic assays targeting virulence genes, especially polymerase chain reaction (PCR), are becoming standard procedure (Iijima et al. 2007).

Diagnosis is currently recommended for cases of persistent diarrhoea, children with severe diarrhoea unresponsive to treatment and immunodeficient patients with moderate to severe diarrhoea, and in epidemic outbreaks of gastroenteritis (Vidal et al. 2005). Methods in molecular biology have progressed and offer significant increases in speed and specificity in identifying micro-organisms according to their specific genetic makeup encoded in the genomic DNA (Horokova et al. 2008). Technologies such as microarrays and PCR are used to explore the global virulence pattern of strains (Wu et al. 2007). However, for developing countries microarray is an expensive method which laboratories cannot afford for routine analysis. M-PCR is a rapid and cost-effective method for screening and identifying DEC. The targets selected for each category were: EHEC (*stx1*, *stx2* and *eaeA*); Atypical EPEC (*eaeA*) and Typical EPEC (*bfp*); ETEC (*st* and *lt*); EIEC (*ial*); EAEC (*eagg*); Commensal *E. coli* (*mdh*); *E. coli* toxin (*astA*) and, for the external control, *gapdh*.

The major obstacle to using PCR for the detection and identification of pathogenic organisms from clinical or environmental water samples is the presence of substances that are inhibitory to PCR such as humic substances (Shieh et al. 1995; Wilson 1997). In order to monitor PCR inhibition sufficient laboratory controls are required in the m-PCR. The majority of published studies report the addition of 16s rRNA gene as the internal control to monitor for false negative results in m-PCR (Sabat et al. 2000; Grape et al. 2007). However, these are not sufficient to monitor false negative results for *E. coli* specifically, since 16s rRNA is amplified from the *E. coli* DNA. It would not be possible to determine whether a lack of PCR amplification of 16s rRNA is as a result of PCR inhibition in the sample or is because there is no *E. coli* in the sample. As reported by Hartman et al. (2005), the high level of PCR sensitivity creates an elevated risk of false positive and negative results.

## Methodology

The aim of this study was to develop a single-step multiplex polymerase chain reaction (m-PCR) that distinguishes selected *E. coli* patho-types. Internal controls were included to monitor inhibition in each sample thereby indicating false positive or false negative results.

### Growth and maintenance of bacterial strains

Thirty-eight bacterial strains, which included commensal and pathogenic *E. coli* strains, *Shigella* spp., *Salmonella* spp., *Vibrio* spp. [obtained from National Health laboratory services (NHLS); (Table 1)] and other strains of the Enterobacteriaceae family such as *Klebsiella* spp., *Aeromonas* spp., *Pseudomonas aeruginosa*, *Bacillus subtilis*, *Bacillus cereus*, *Enterococcus* spp. and *Morganella morganni* (obtained from undergraduate practical laboratory) were cultured on Plate Count Agar (PCA) (Oxoid, UK) and incubated under aerobic conditions at 37 °C for 16 h. Single colonies were enriched in nutrient broth (Oxoid, UK) and incubated under aerobic conditions at 37 °C for 16 h. The commensal *E. coli* strain was used as the positive control. *Klebsiella pneumoniae* (KLEPN 01) and *Pseudomonas aeruginosa* (PSEAE 01) were used as the negative controls for the Colilert^®^ Quanti-Trays^®^/2000.

**Table 1:** Bacterial strains used in molecular characterisation

Bacterial strain	Reference nr	Genes present
*Escherichia coli* (Commensal)^a^	ATCC 25922	*mdh*
Enterohaemorrhagic (EHEC)	ESCCO 21^b^	*mdh, stx1*, *stx2* and *eaeA*
Enteroinvasive (EIEC)	ESCCOS ATCC 43893^b^	*mdh* and *ial*
Enterotoxigenic (ETEC)	ESCCO 22^b^	*mdh, lt* and *st*
Enteropathogenic (EPEC)	S-ESCCO 16 Pl^b^	*mdh, eaeA, bfp*
Enteroaggregative (EAEC)	ESCCO 14^b^	*mdh* and *eagg*

^a^Environmental isolate confirmed by API 20E (OMNIMED^®^) and PCR as commensal *E. coli*

^b^Strains purchased from National Health Laboratory Services (NHLS)

### M-PCR testing on enriched environmental water samples and isolates

Once the m-PCR was developed it was tested on clinical, environmental isolates and environmental water samples.

### Microbial analysis

#### Clinical isolates

239 clinical isolates were obtained from Ampath Laboratory (Pretoria). Single colonies which were confirmed *E. coli* positive by Ampath Laboratory were enriched as described above (growth and maintenance).

#### Environmental isolates

171 environmental water samples (container water, toilet seats, borehole, stream, river) were collected in 1 l sampling bottles and stored at 4 °C on route to the laboratory. The water samples (100 ml) were filtered onto 0.45 μm gridded nitro-cellulose membranes (NC) (Merck, Germany) using the standard membrane filtration technique, placed onto *E. coli*/Coliform Chromogenic Media (Oxoid, UK) and incubated under aerobic conditions at 37 °C for 16 h (Standard Methods 2005). Single colonies that appear purple on the selective *E. coli* media were enriched as described above in growth and maintenance.

#### Environmental water samples

291 water samples (Waste water: upstream, downstream and final effluent) were collected in 1 l sampling bottles and stored at 4 °C on route to the laboratory. The water samples were immediately analysed upon arrival at the laboratory for bacterial quality using the Colilert^®^ Quanti-Tray^®^/2000 system (IDDEX). Enumeration of *E. coli* from water was done using 100 ml water according to the manufacturer’s instructions. The Quanti-Trays^®^ were incubated for 18 h at 35 °C. After incubation, the Quanti-Trays^®^/2000 were examined under long wave (366 nm) ultraviolet light, and wells that turned both yellow and fluoresced were counted as *E. coli* positive (IDDEX).

### DNA extraction

#### Clinical and environmental isolates

2 ml of the enriched single colony was centrifuged for 2 min at 13,000×*g* to pellet the cells and the supernatant was discarded. DNA was extracted from the collected bacterial cells using the silica/guanidium thiocyanate method reported by Boom et al. (1990) as well as adaptations of spin columns reported on by Borodina et al. (2003). The adjustments included the addition of 250 µl 100 % ethanol to the lysis buffer to enhance the binding of DNA to the Celite. The Celite containing the bound DNA was loaded onto a DNA binding membrane (Borodina et al. 2003) in the spin columns. DNA was eluted with 100 µl Qiagen elution buffer (Southern Cross Biotechnology^®^) [Omar et al. (2010)]. The extracted DNA was used as a template in all PCR reactions.

#### Colilert^®^ Quanti-Trays^®^/2000 system

A total of 2 ml of the media was removed from up to ten positive *E. coli* wells of the Colilert^®^ Quanti-Trays^®^/2000 using sterile 1 ml Neomedic disposable syringes with mounted needle (Kendon Medical Supplies) and aliquoted into 2 ml sterile Eppendorf tubes. The tubes were centrifuged for 2 min at 13,000×*g* to pellet the cells and the supernatant discarded. DNA was extracted from the collected bacterial cells as explained above and as reported by Omar et al. (2010). The extracted DNA was used as a template in all PCR reactions.

### Multiplex polymerase chain reaction (m-PCR)

All m-PCR reactions were performed in a Biorad Mycycler™ thermal cycler in a total reaction volume of 20 μl. A hotstart multiplex PCR kit (Qiagen^®^) was used for the m-PCR protocol. Each reaction consisted of 1X Qiagen^®^ PCR multiplex mix (containing HotstartTaq^®^ DNA polymerase, multiplex PCR buffer and dNTP mix); 2 μl of the primer mixture [0.1 μM of *mdh* and *lt* primers [Forward (F) and reverse (R)], 0.2 μM of *ial*, *eagg* primers, *astA* primers, *bfp* primers and *gapdh* primers (F and R), 0.3 μM of *eaeA* and *stx2* primers (F and R), 0.5 μm of *stx1* and *st* primers [F and R (Table 2)]; 2 μl of sample DNA, 1 μl of *gapdh* cDNA and 5 μl PCR grade water. The reactions were subjected to an initial activation step at 95 °C for 15 min, followed by 35 cycles consisting of denaturing at 94 °C for 45 s, annealing at 55 °C for 45 s, extension at 68 °C for 2 min and final elongation at 72 °C for 5 min.

**Table 2:** Primers used in the m-PCR reaction

Pathogen	Primer	Sequence(5′-3′)	Size (bp)	Conc. (µM)	Reference
*E. coli*	*mdh (F)*	GGT ATG GAT CGT TCC GAC CT	304	0.1	Tarr et al. (2002)
	*mdh (R)*	GGC AGA ATG GTA ACA CCA GAG T			
EIEC	*ial (F)*	GGT ATG ATG ATG ATG AGT CCA	650	0.2	López-Saucedo et al. (2003)
	*ial (R)*	GGA GGC CAA CAA TTA TTT CC			
EHEC/Atypical EPEC	*eaeA (F)*	CTG AAC GGC GAT TAC GCG AA	917	0.3	Aranda et al. (2004)
	*eaeA (R)*	CCA GAC GAT ACG ATC CAG			
Typical EPEC	*bfpA (F)*	AAT GGT GCT TGC GCT TGC TGC	410	0.3	Aranda et al. (2004)
	*bfpM (R)*	TAT TAA CAC CGT AGC CTT TCG CTG AAG TAC CT			From this study
EAEC	*eagg (F)*	AGA CTC TGG CGA AAG ACT GTA TC	194	0.2	Pass et al. (2000)
	*eagg (R)*	ATG GCT GTC TGT AAT AGA TGA GAA C			
EHEC	*stx1 (F)*	ACA CTG GAT GAT CTC AGT GG	614	0.5	Moses et al. (2006)
	*stx1 (R)*	CTG AAT CCC CCT CCA TTA TG			
	*stx2 (F)*	CCA TGA CAA CGG ACA GCA GTT	779	0.3	Moses et al. (2006)
	*stx2 (R)*	CCT GTC AAC TGA GCA CTT TG			
ETEC	*lt (F)*	GGC GAC AGA TTA TAC CGT GC	360	0.1	Pass et al. (2000)
	*lt (R)*	CGG TCT CTA TAT TCC CTG TT			
	*st (F)*	TTT CCC CTC TTT TAG TCA GTC AAC TG	160	0.5	Pass et al. (2000)
	*st (R)*	GGC AGG ATT ACA ACA AAG TTC ACA			
*E. coli* toxin	*astA (F)*	GCC ATC AAC ACA GTA TAT CC	106	0.3	Kimata et al. (2005)
	*astA (R)*	GAG TGA CGG CTT TGT AGT C			
External control	*gapdh (F)*	GAG TCA ACG GAT TTG GTC GT	238	0.3	Mbene et al. (2009)
	*gapdh (R)*	TTG ATT TTG GAG GGA TCT CG			

NB: *F* forward primer, *R* reverse primer

DNA was visualised using a 2.5 % (w/v) agarose gel in TAE buffer (40 mmol l^−1^ Tris acetate; 2 mmol l^−1^ EDTA, pH 8.3) with 0.5 μg ml^−1^ ethidium bromide. Electrophoresis was done for 1–2 h in electric field strength of 8 V cm^−1^ gel and the DNA visualized with UV light (Syngene, UK). This procedure was followed for all the experiments except where stated differently. The relative sizes of the DNA fragments were estimated by comparing their electrophoretic mobility with that of the standards run with the samples on each gel, either a 1 kB or 100 bp markers (Fermentas, US).

### Specificity of the m-PCR

The specificity of the m-PCR was assessed by testing 38 bacterial strains which included commensal and pathogenic *E. coli* strains, *Shigella* spp., *Salmonella* spp. and serovar, *Vibrio* spp. and other strains of the Enterobacteriaceae family such as *Klebsiella* spp., *Aeromonas* spp., *Pseudomonas aeruginosa*, *Bacillus subtilis*, *Bacillus cereus, Enterococcus* spp. and *Morganella morgannii* (Table 3).

**Table 3:** Specificity of the m-PCR

Bacterial strain	Source	Genes										
		*mdh*	*eaeA*	*bfp*	*stx1*	*stx2*	*ial*	*lt*	*gapdh*	*st*	*eagg*	*astA*
Commensal *E. coli*	NHLS	+	−	−	−		−	−	+	−	−	−
Enterohaemorrhagic *E. coli*	NHLS	+	+	−	+	+	−	−	+	−	−	−
Enteropathogenic *E. coli*	NHLS	+	+	+	−	−	−	−	+	−	−	+
Enteroaggregative *E. coli*	NHLS	+	−	−	−	−	−	−	+	−	+	−
Enterotoxigenic *E. coli*	NHLS	+	−	−	−	−	−	+	+	+	−	−
Enteroinvasive *E. coli*	NHLS	+	−	−	−	−	+	−	+	−	−	−
*Shigella dysenteriae* serovar type 1	NHLS	+	−	−	−	−	+	−	+	−	−	−
*Shigella dysenteriae* serovar type 2	NHLS	+	−	−	−	−	+	−	+	−	−	−
*Shigella boydii* serovar B	NHLS	−	−	−	−	−	−	−	+	−	−	−
*Shigella flexneri*	NHLS	+	−	−	−	−	−	−	+	−	−	−
*Shigella sonnei*	NHLS	+	+	−	−	−	+	−	+	−	−	−
*Vibrio cholerae* non-O1	NHLS	−	−	−	−	−	−	−	+	−	−	−
*Vibrio cholerae* O1	NTCC	−	−	−	−	−	−	−	+	−	−	−
*Vibrio cholerae* O1	NTCC	−	−	−	−	−	−	−	+	−	−	−
*Vibrio parahaemolyticus*	NHLS	−	−	−	−	−	−	−	+	−	−	−
*Vibrio parahaemolyticus*	NCTC	−	−	−	−	−	−	−	+	−	−	−
*Vibrio cholerae* O139	NHLS	−	−	−	−	−	−	−	+	−	−	−
*Vibrio cholerae* Ogawa	NHLS	−	−	−	−	−	−	−	+	−	−	−
*Vibrio mimicus*	NHLS	−	−	−	−	−	−	−	+	−	−	−
*Vibrio fluvialis*	NCTC	−	−	−	−	−	−	−	+	−	−	−
*Vibrio furnissii*	ATCC	−	−	−	−	−	−	−	+	−	−	−
*Salmonella enterica* serovar Typhi salty O1	NHLS	−	−	−	−	−	−	−	+	−	−	−
*Salmonella enterica* serovar Typhimurium saltm O1	NHLS	−	−	−	−	−	−	−	+	−	−	−
*Salmonella enterica* serovar Typhimurium saltm O2	NHLS	−	−	−	−	−	−	−	+	−	−	−
*Salmonella enterica* serovar Typhi salty O2	NHLS	−	−	−	−	−	−	−	+	−	−	−
*Salmonella enterica* serovar Paratyphi	NHLS	−	−	−	−	−	−	−	+	−	−	−
*Salmonella enterica* serovar Paratyphi A	NHLS	−	−	−	−	−	−	−	+	−	−	−
*Salmonella enterica* serovar Paratyphi C	NHLS	−	−	−	−	−	−	−	+	−	−	−
*Salmonella* Gallinarum	NHLS	−	−	−	−	−	−	−	+	−	−	−
*Salmonella enterica* serovar Enteritidis	NHLS	−	−	−	−	−	−	−	+	−	−	−
*Pseudomonas aeruginosa*	NHLS	−	−	−	−	−	−	−	+	−	−	−
*Klebsiella pneumonia*	NHLS	−	−	−	−	−	−	−	+	−	−	−
*Bacillus subtilis*	NHLS	−	−	−	−	−	−	−	+	−	−	−
*Bacillus cereus*	NHLS	−	−	−	−	−	−	−	+	−	−	−
*Aeromonas veronii*	ATCC	−	−	−	−	−	−	−	+	−	−	−
*Enterococcus faecium*	NHLS	−	−	−	−	−	−	−	+	−	−	−
*Enterococcus faecalis*	NHLS	−	−	−	−	−	−	−	+	−	−	−
*Morganella morgannii*	NHLS	−	−	−	−	−	−	−	+	−	−	−

## Results and discussion

**Fig. 1:**
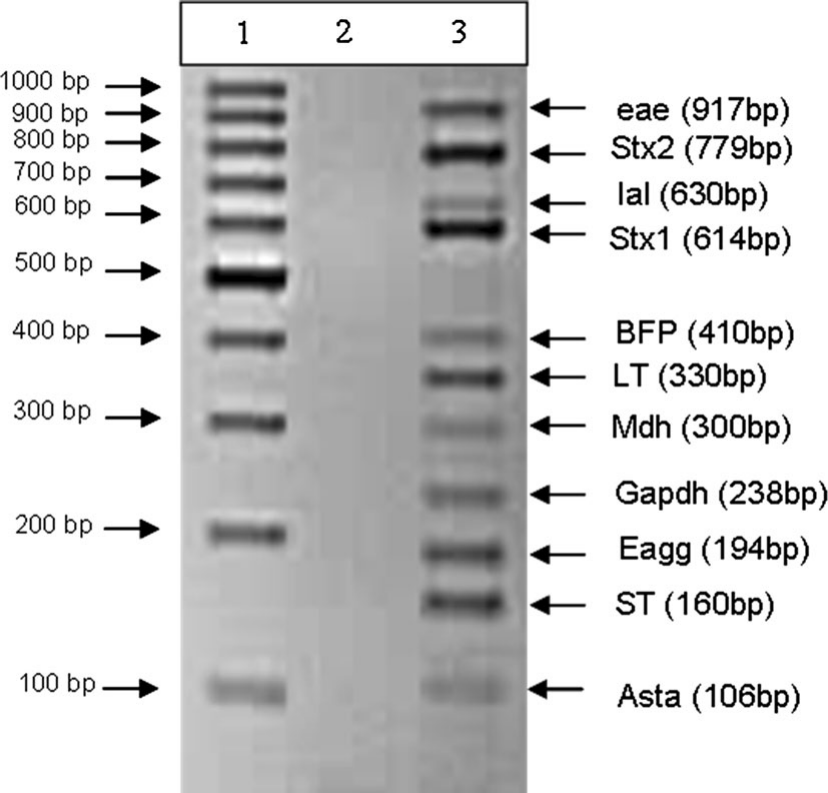
Agarose gel of the PCR products obtained for the *E. coli* multiplex PCR (*lane* *2*). No template control (NTC) in (*lane 2*). The molecular weight marker is shown in (*lane 1*)

The main challenge of designing a multiplex PCR is the possibility of primer dimers and non-specific results which is a risk for false positive and negative results. Therefore, it is necessary to design and include primers with close annealing temperatures and to begin the program with a hotstart as reported by Vidal et al. (2005). The effect of the wide temperature range is overcome by the addition of Q-solution that is supplied by the manufacturer and that can be included with the enzyme. A wide variety of temperatures were tested before the final version of the multiplex PCR was optimized and tested. The results confirm that the single m-PCR was successfully compiled to detect all of the targeted genes in a single reaction even though primers with different melting temperatures ranging from 50 to 73 °C were used (Fig. 1). The PCR amplicons were confirmed as the correct target gene by sequencing (data not shown) showing the specific amplification of the genes in a mixture of DEC.

### Specificity of the m-PCR

The specificity of the m-PCR was tested on 38 laboratory bacterial strains. Specificity was stated by Aldrich and Griffith (2003) as ‘the ability of the assay to detect a unique event to the exclusion of all other events’; that is, to what extent can the assay detect a specific pathobiologic effect that will exclude all other similar pathobiologic effects. Positive PCR results were only obtained for the *E. coli* and *Shigella* strains (Table 3). However, the *mdh* gene was not detected for *Shigella boydii* serotype B. Boerlin et al. (1999) state that *Shigella* is similar to EIEC and the *stx1* is almost identical to the shiga toxin of *Shigella dysenteriae* in amino acid sequence and cannot be distinguished from serologically, yet *ial* and *eaeA* were detected for *Shigella sonnei*. No positive PCR results were obtained for the DNA from the other bacterial strains tested. Specific genes were detected for each patho-type as indicated in Table 1; there was no cross reactivity of genes between patho-types. No false positives and no PCR inhibition were obtained due to the external control *gapdh* gene that was detected in 100 % (38/38) of the samples.

### Application

**Fig. 2:**
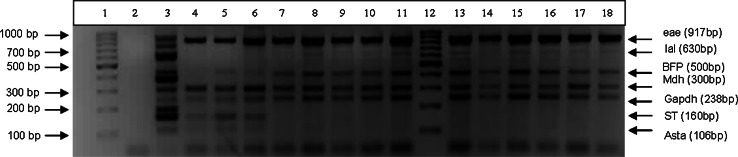
Agarose gel of the PCR products obtained from samples (*lane 4*–*11*, *13*–*18*). No template control (NTC) in (*lane 2*). The molecular weight marker is shown in (*lane 1* and *12*). The positive reference control is shown in (*lane* *3*)

A total of 701 samples were analysed, samples composed of 239 clinical isolates, 171 environmental water isolates and 291 samples from the Colilert^®^ Quanti-Tray^®^/2000 (Fig. 2); these samples were obtained from various provinces in South Africa. Isolates and water samples were subjected to the protocols described in the methodology, with 100 % (171/171) of environmental water isolates, 85 % (202/239) of the clinical isolates and 100 % (291/291) of the water samples testing positive for the *mdh* house-keeping gene (Fig. 2). For the 15 % (37/239) of clinical isolates in which the *mdh* gene was not detected, it is possible that these do not contain the malate dehydrogenase but the malic acid dehydrogenase gene, which is also a housekeeping enzyme of the citric acid cycle (Hsu and Tsen 2001). When the study was initiated Tarr et al. (2002) article was used, who included the malate dehydrogenase gene and indicated in their tests positive results for all the *E. coli* strains tested. Based on this the *mdh* gene was used as a control to confirm the microbiology results in case no pathogenic genes tested for were detected. It is only later that for a separate study the malic acid dehydrogenase gene was tested (also referred to as *mdh* by Hsu and Tsen 2001) that not all the *E. coli* strains present in the samples tested positive. The reason could be that the original work by the authors were done on strains that could not be present in South Africa or that we have strains that have different genetic characteristics. No false positives and no PCR inhibition were indicated in the m-PCR as the external control gene (*gapdh*) was detected in 100 % (701/701) of the samples. A supposedly negative test result for an infectious agent can influence therapeutic decisions, such as withholding antibiotic and antiviral drugs (Cone et al. 1992; Hartman et al. 2005). Therefore, the additions of the internal and external controls are important to ensure that there are no PCR inhibitors in the reaction as well as to validate the accuracy of the PCR in distinguishing false negative from true negative PCR results.

#### Environmental water isolates

Of the *E. coli* positive environmental water isolates (171) tested, *eagg* gene (EAEC), *ial* gene (EIEC), *st* and *lt* genes (ETEC), *stx1* gene and *stx2* gene, *eaeA* gene (EHEC, Atypical-EPEC) tested positive (see Table 4 for the percentages of each gene). Positive gene combinations detected for *eaeA* and *stx1* 2.3, 0.6 % combination of *eaeA*, *stx1* and *stx2* (EHEC). Literature states that *stx1* and/or *stx2* can be detected individually or in combination due to being phage-encoded (Müller et al. 2001; Contreras et al. 2011; Feng et al. 2011). To discriminate between typical and atypical EPEC, 29.8 % tested positive for the *eaeA* and *bfp* gene (Typical-EPEC), 3.5 % *bfp* gene (Typical-EPEC) and 27 % *eaeA* gene (Atypical EPEC). For the *astA* gene (*E. coli* toxin) 25 % was detected without the combination of the virulence genes. The distribution of the *astA* toxin gene combined with the virulence genes is indicated in Table 6. Interesting results was 2.3 % combination of *eaeA* and *ial* as well as combination of *eaeA* and *eagg* 8.7 % (Table 5). Literature reports gene coding of *eaeA* with EHEC and EPEC (Presterl et al. 2003; Müller et al. 2001; Aranda et al. 2004; Moses et al. 2006) but not with *eagg* and *ial*. Published reports have described *eaeA* as the bacterial outer membrane protein intimin, which is essential in organizing host cytoskeletal rearrangements and generating the pedestal-like structure in which the bacteria reside. Intimin is required for full bacterial virulence and its expression is regulated by the *per* regulon. The *per* regulon comprises four reading frames (*perA*, *B*, *C* and *D*), and maximal expression requires all four gene products, however, expression of *perC* alone can induce intimin expression (Kenny et al. 1997). The question is has intimin been expressed from EAEC and EIEC? Or as Chen and Dubnau (2004) reported that DNA can be transferred from one organism to another via conjugation. They also reported DNA can be actively secreted by viable organisms. Hacker and Kaper (2000) reported that free DNA released from dead bacteria can be taken up by bacteria in the environment via natural transformation and may carry pathogenicity islands (PAIs). The majority of PAIs are located on the chromosome, but can also be part of bacterial plasmids and phages. More research has to be conducted to determine these gene-coding combinations.

**Table 4:**
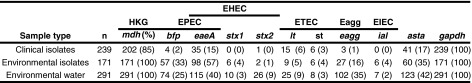
PCR results obtained from the single isolates of the clinical and environmental isolates and water samples from the Colilert^®^ Quanti-Tray^®^/2000

**Table 5:** Gene combinations from clinical and environmental isolates

Patho-type	Gene combinations	Clinical isolates (n)	Environmental isolates (n)	References
Atypical EPEC	*eaeA*	32	46	Aranda et al. (2004); Botkin et al. (2012)
EHEC	*eaeA* + *stx1*	0	4	Müller et al. (2001); Contreras et al. (2011); Feng et al. (2011)
	*eaeA* + *stx2*	1	0	
	*eaeA* + *stx1* +*stx2*	0	1	
	*stx1*	0	1	
	*stx2*	0	1	
Typical EPEC	*eaeA* + *bfp*	2	51	Kaper et al. (2004);
	*bfb*	2	6	Botkin et al. (2012)
ETEC	*lt* + *st*	0	3	Presterl et al. (2003)
	*lt*	8	6	
	*st*	6	3	
	*lt* + *eagg*	1	0	
	*eaeA* + *ial*	0	4	
	*eaeA* + *eagg*	0	15	

#### Clinical isolates

Of the clinical isolates (239) tested, *eagg* (EAEC), *lt* and *st* (ETEC), *eaeA* and *stx2* (EHEC, Atypical-EPEC) tested positive (Table 4). Positive gene combinations were detected for 0.8 % *eaeA* and *bfp* (Typical-EPEC), 0.8 % *bfp* (Typical-EPEC) and 13.4 % *eaeA* gene (Atypical EPEC), 17 % *astA* (*E. coli* toxin). The significance of differentiating between typical and atypical EPEC is that atypical EPEC are more frequently isolated from diarrhoea cases than typical EPEC. However, while typical EPEC dominates in developing countries, atypical EPEC has also been shown to cause large outbreaks involving both children and adults (Kaper et al. 2004). For the *astA* gene (*E. coli* toxin) 78 % was detected without the combination of the virulence genes. The distribution of the *astA* toxin gene combined with the virulence genes is indicated in Table 6. This result is very important: Hidaka et al. (2009) reported that a 1996 outbreak of gastrointestinal illness was caused by *E. coli* 0166:H15 which possessed no enteropathogenicity-associated genes other than the *astA* gene. The *astA* gene was first identified in EAEC as a structural gene that encodes a distinct low-molecular-weight putative enterotoxin (Yatsuyanagi et al. 2003). Reports from Soto et al. (2009) indicate that the enteroaggregative heat stable toxin 1 (EAST-1) is encoded by the *astA* gene. This toxin is thought to play a role in EAEC pathogenicity. The toxin binds to the receptor and activates guanylate cyclise, which stimulates production of cyclic GMP (cGMP). High levels of cGMP in the cell inhibit the Na/Cl co-transport system, reduce the absorption of electrolytes and water from the intestine at villus tips and result in an elevated secretion of Cl^−^ and water in crypt cells. The role of this toxin in the development of diarrhoea has yet to be defined (Soto et al. 2009). However, recently the *astA* gene has been detected not only in EAEC but also in EPEC, atypical EPEC, ETEC and EIEC strains (Yatsuyanagi et al. 2003). As discussed above, an interesting gene-coding combination was detected in the clinical isolates, 0.4 % *lt* and *eagg* genes.

**Table 6:**
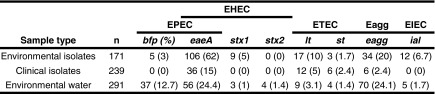
Distribution of the *astA* toxin gene combined with the virulence genes for isolates, environmental isolates and water samples

#### Environmental water samples

Of the *E. coli* positive environmental water samples from the Colilert^®^ Quanti-Tray^®^/2000 (291) tested, presence of *eagg* gene (EAEC), *ial* gene (EIEC), *lt* gene and *st* gene (ETEC) tested positive (Table 4). Positive gene combination detected for 0.3 % of *eaeA* and *stx1*, 5.8 % combination of *eaeA* and *stx2* (EHEC), 3.1 % combination of *eaeA*, *stx1* and *stx2* (EHEC). To discriminate between typical and atypical EPEC 24.1 % tested positive for the *eaeA* and *bfp* gene (Typical-EPEC) and 1.4 % *bfp* gene (Typical-EPEC) 17 % *eaeA* gene (Atypical EPEC). For the *astA* gene (*E. coli* toxin) 40 % was detected without the combination of the virulence genes and the distribution of the *astA* toxin gene combined with the virulence genes are indicated in Table 6.

## Conclusion

Both internal controls for m-PCR were used to monitor PCR inhibition that might occur due to the nature of the samples. The PCR was designed so that the *gapdh* gene would only be amplified in samples where no other PCR products were amplified. All the genes tested for could be detected using m-PCR with no non-specific amplification of genes. Atypical and typical EPEC could be successfully distinguished using single m-PCR reaction. The *astA* toxin gene was detected in both DEC and ComEC samples. Important gene combinations were detected. The m-PCR offers the user a fast and effective method to perform a simultaneous amplification not only for the detection of virulence genes from all categories of diarrhoeagenic *E. coli* (ETEC, typical or atypical EPEC, EIEC, EAEC, EHEC) but also commensal *E. coli* and internal controls to monitor for PCR inhibition. The m-PCR is easy to perform, sensitive, requires minimal specialized equipment or training, and provides same-day results necessary for rapid action in the case of diarrhoeal outbreaks.
